# Plasma *p*‐Tau217 assay performance in Japan: from preclinical/prodromal cohort to DMT registry

**DOI:** 10.1002/alz70856_097513

**Published:** 2025-12-24

**Authors:** Takeshi Iwatsubo

**Affiliations:** ^1^ Department of Neuropathology, Graduate School of Medicine, The University of Tokyo, Bunkyo‐ku, Tokyo, Japan

## Abstract

**Background:**

There is an urgent need to determine the utility of plasma biomarkers, particularly tau phosphorylated at threonine 217 (*p*‐tau217), in predicting amyloid PET positivity and monitoring the efficacy of disease‐modifying therapies (DMT) in the early stages of Alzheimer's disease (AD).

**Method:**

Participants in the Japanese trial‐ready cohort for preclinical/prodromal AD (J‐TRC), which included 474 non‐demented individuals (CDR 0: 331, CDR 0.5: 143), were assessed by amyloid PET (florbetapir or flutemetamol) and plasma biomarker assays. Plasma Aβ42 and Aβ40 were measured by IP mass spectrometry (Shimadzu), and *p*‐tau217 was measured by immunoassay on the Meso Scale Discovery platform (Eli Lilly). The study was conducted by collaborative groups from the J‐TRC and the AD DMT Registry.

**Result:**

Aβ‐PET was abnormal in 81 participants. High accuracy in detecting an abnormal Aβ‐PET scan was achieved by plasma *p*‐tau217 (AUC = 0.913) or Aβ42/40 (AUC = 0.856), which was improved by combining plasma biomarkers and by including clinical information in the models. The highest AUCs were observed for the models combining *p*‐tau217/Aβ42 ratio, APOE, age, sex in the whole cohort (AUC = 0.936), *p*‐tau217, Aβ42/Aβ40 ratio, APOE, age, sex in the CDR 0 group (AUC = 0.948), and *p*‐tau217/Aβ42 ratio, APOE, age, sex in the CDR 0.5 group (AUC = 0.955), respectively.

**Conclusion:**

These results indicate that plasma *p*‐tau217 alone, or combinations of plasma Aβ biomarkers and *p*‐tau217, are effective in predicting Aβ‐PET positivity in preclinical and prodromal AD. The ability of the *p*‐tau217 plasma assays to prescreen eligible patients and monitor the efficacy of anti‐Aβ antibodies and other DMTs is also being tested in a real‐world setting in the Japanese early symptomatic AD patient registry.

